# Effect of a Novel, Energy-Dense, Low-Volume Nutritional Food in the Treatment of Superior Mesenteric Artery Syndrome

**DOI:** 10.7759/cureus.15243

**Published:** 2021-05-25

**Authors:** Tetsuro Akashi, Risa Hashimoto, Akihiro Funakoshi

**Affiliations:** 1 Department of Internal Medicine, Saiseikai Fukuoka General Hospital, Fukuoka, JPN; 2 Department of Internal Medicine, Saku Hospital, Fukuoka, JPN

**Keywords:** superior mesenteric artery syndrome, oral nutritional supplements, energy intake, enteral nutrition, tube feeding

## Abstract

Superior mesenteric artery syndrome (SMAS) is an intermittent or persistent passage obstruction that occurs in the third portion of the duodenum between the aorta and the superior mesenteric artery. After symptoms stabilize, the nutritional intake is started by ingesting a small amount. Recently, an energy-dense, low-volume nutritional food, Terumeal uplead^®^ (Terumo Corporation, Tokyo, Japan) with an energy density of 4.0 kcal/mL, was launched. We report a case of a postoperative SMAS patient who was successfully treated using Terumeal uplead^®^ through gastrostomy. An 83-year-old man who developed adhesive intestinal obstruction underwent right hemicolectomy, lysis of adhesion, and partial small bowel resection. Gastric distension persisted after surgery; thus, gastrostomy was performed for decompression and enteral nutrition on the 21st postoperative day, and enteral feeding was started on the 23rd postoperative day. However, fluoroscopy showed obstruction in the third portion of the duodenum, which was considered to be SMAS. To reduce the administration volume, enteral nutrition was replaced with Terumeal uplead^®^ from the 28th postoperative day (intermittent administration thrice a day, 300 mL, 1,200 kcal per day). From the 34th postoperative day, the gastrostomy tube was clamped for two hours after administration, and no drainage was observed. Oral intake was resumed from the 36th postoperative day, and it was used in combination with enteral nutrition. Three months later, the patient was discharged home and continued oral ingestion with occasional decompression from the gastrostomy tube. Thus, Terumeal uplead^®^ may be useful during the conservative treatment of SMAS by initiation with small amounts.

## Introduction

Superior mesenteric artery syndrome (SMAS) is a relatively rare disease that causes compression of the third portion of the duodenum between the superior mesenteric artery and the aorta [1]. Its symptoms include vomiting, nausea, abdominal pain, bloating, and anorexia [1]. The pathogenic mechanisms underlying the SMAS include both congenital (high insertion of the ligament of Treitz, intestinal malrotation, peritoneal adhesions, low origin of the superior mesenteric artery, and increased lumbar lordosis) and acquired (rapid weight loss, long-term bed rest, abdominal surgery, retroperitoneal mass, and wearing a corset) factors [2]. The treatment of SMAS focuses on increasing the mass of retroperitoneal fat and preventing recurrence, but surgery is considered in refractory cases. Jejunal nutrition is a conservative treatment, wherein the tip of the feeding tube exists beyond the obstruction of the duodenum; it is considered to be successful for administering sufficient nutrition [3-7]. However, guiding the nasal tube or the gastrostomy tube to the jejunum is complicated, and there is a concern that enteral nutrition may not be started immediately in some cases.

Recently, an energy-dense, low-volume nutritional food, Terumeal uplead® (Terumo Corporation, Tokyo, Japan) with an energy density of 4.0 kcal/mL, has been launched. Terumeal uplead® provides high energy even when consumed in small amounts. It is useful when one cannot consume large amounts of food at once. Treatment of SMAS includes increasing the mass of retroperitoneal fat by starting a small meal after the symptoms stabilize [8]. Therefore, Terumeal uplead® may be considered as a nutritional food suitable for SMAS treatment, which may be administered transgastrically. We present an improved case of a postoperative SMAS patient who received Terumeal uplead® from the gastrostomy tube. The Ethics Committee of Saiseikai Fukuoka General Hospital approved this case report and waived the requirement of informed consent. The treatment protocol complied with the principles of the Declaration of Helsinki.

## Case presentation

**Figure 1:**
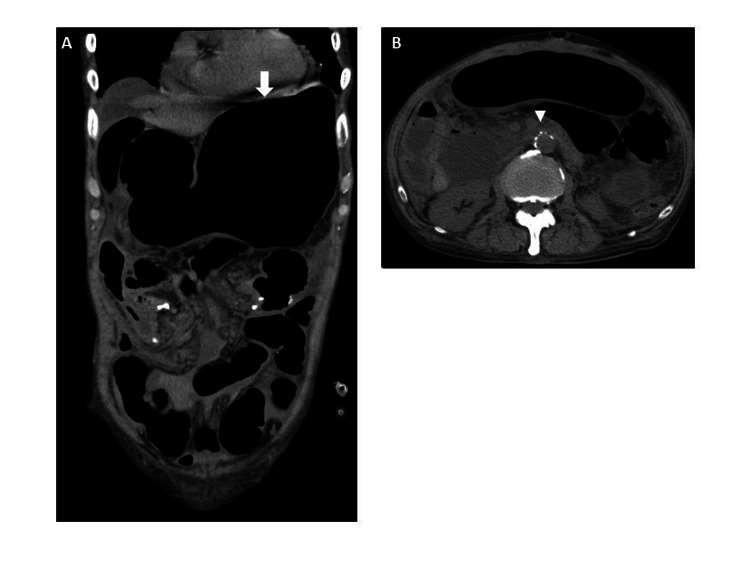
Findings of the CT scan performed on the seventh postoperative day (A) Coronal CT findings showed that the stomach (white arrow) was highly dilated, but no obstruction was observed in the pelvic small intestine and colon. (B) Axial CT findings showed that the aorta-mesenteric distance (white arrowhead) was 4.4 mm. CT, computed tomography

**Figure 2:**
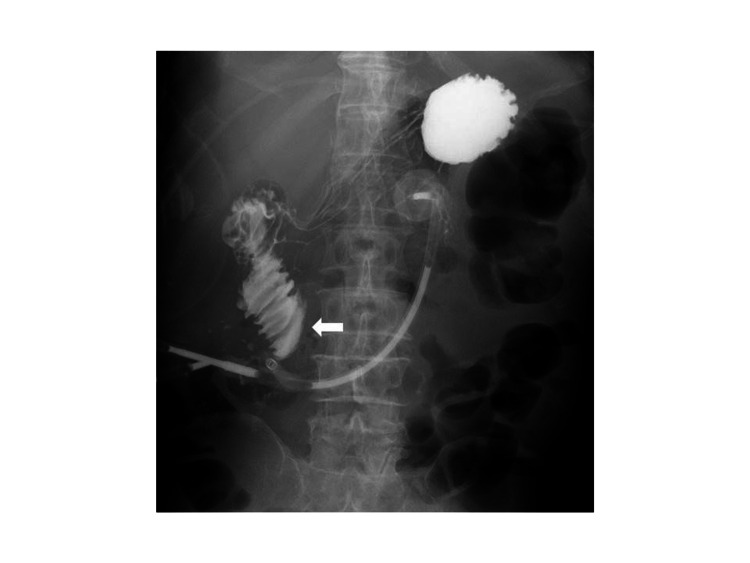
Findings of the fluoroscopy performed on the 23rd postoperative day Fluoroscopy revealed that the contrast medium stagnated in the third part of the duodenum (white arrowhead), and when the posture was changed, the contrast medium flowed into the small intestine.

An 83-year-old man had undergone surgery for cecal volvulus and intestinal obstruction four months ago. He then had an adhesive intestinal obstruction that developed three months after the first surgery, but conservative treatment was not successful; thus, he underwent right hemicolectomy, lysis of adhesion, and partial small bowel resection. The placement of the ileus tube was continued after the operation. A large amount of watery feces was observed on the fifth postoperative day, and the ileus tube was removed on the sixth postoperative day. On the seventh postoperative day, a high degree of gastric dilatation was observed, and decompression was performed using a nasogastric tube. Computed tomography (CT) scan showed no occlusion in the pelvic small intestine and colon, and the aorta-mesenteric distance was 4.4 mm (Figure 1). The glutamine preparation was administered from the eighth postoperative day onward, but the gastric dilation continued, and a gastrostomy was performed on the 21st postoperative day for decompression and enteral nutrition. From the 23rd postoperative day, enteral nutrition was started through the gastrostomy (intermittent administration thrice a day, 800 mL, 1,600 kcal per day). However, the fluoroscopy revealed that the contrast medium stagnated in the third part of the duodenum, and when the body position was changed, the contrast medium managed to flow into the small intestine (Figure 2). These findings indicated the occurrence of SMAS. The gastrostomy tube was appropriately opened and depressurized, and a drainage of 500-1,500 mL/day was observed. To reduce the administration volume, enteral nutrition was changed to energy-dense, low-volume nutritional food, Terumeal uplead®, from the 28th postoperative day (intermittent administration thrice a day, 300 mL, 1,200 kcal per day). From the 34th postoperative day onward, the gastrostomy tube was clamped for two hours after the administration, and no fluid discharge was observed. The swallowing function also recovered. The oral intake was resumed from the 36th postoperative day, and it was combined with enteral nutrition. The patient was transferred to another hospital on the 49th postoperative day for continuous medical treatment. Three months later, he was discharged home and continued with oral consumption along with appropriate reduction of the intragastric pressure from the gastrostomy tube. One year after the surgery, he has become accustomed to handling the gastrostomy tube and is continuing palliative treatment.

## Discussion

In SMAS, the restoration of the retroperitoneal fat by repeated oral administration of small amounts of food in the left lateral or prone position after gastric decompression effectively relieves the obstruction [8]. In case the conservative treatment does not improve the condition or recurrence occurs, surgical treatment is opted. Nutritional therapy is important because many patients with weight loss and long-term bed-ridden conditions have the risk from surgery. Nutritional support using transjejunal feeding past the point of obstruction has been reported to be useful as an active conservative treatment [3-8]. However, transjejunal feeding is invasive and, sometimes, a technically difficult procedure [9-11]; moreover, there is concern that enteral nutrition may not be immediately initiated.

In our case, the patient with postoperative SMAS was fed transgastrically with an energy-dense, low-volume nutritional food, and improvement in symptoms was observed. Although transjejunal feeding was also considered, percutaneous endoscopic gastrostomy with jejunal extension required further endoscopic procedures and fluoroscopy, and the procedure would have been invasive; thus, we first administered enteral nutrition using the recently released energy-dense nutritional food.

Enteral nutrition can be provided in the form of liquid supplements. For patients who are unable to take enough nutritional supplements orally, they are fed using an enteral tube into the stomach or small intestine [9]. The energy density of commercialized formulations is approximately in the range of 0.5-2.4 kcal/mL [9,12]. Enteral nutrition is an effective strategy for treating and avoiding malnutrition and is given as an oral nutritional supplement (ONS) when the patient is able to take it orally. A positive correlation has been reported between the compliance and energy density of ONS [12-13].

Terumeal uplead® is an energy-dense, low-volume nutritional food that was launched in 2017. It has an osmotic pressure of 420 mOsm/L, a caloric density of 4 kcal/mL, and viscosity of approximately 10,000 mPa s. The energy distribution of the formula (units/100 mL) is 14 g of protein, 21.6 g of fat, and 37.4 g of carbohydrates. The fat comprises 41% saturated fatty acids, 35.6% monounsaturated fatty acids, and 23.4% polyunsaturated fatty acids. In addition, 30% of fat contains medium-chain fatty acids. Terumeal uplead® is often used as an ONS, and in case it cannot be taken orally, it is administered using a large-diameter gastrostomy tube rather than a nasogastric tube owing to its higher viscosity than the liquid enteral formula. Like other ONSs, Terumeal uplead® is often used when oral intake is not sufficient, and it is expected to improve compliance owing to its high-energy density in low volume. In our case, it was possible to administer a lesser volume (100 mL each time) than the conventional volume (200 or 400 mL each time) of liquid enteral formula. It was considered that a sufficient volume could be administered, without exacerbation of the symptoms, even from the gastrostomy; therefore, the administration was continued without worsening of the symptoms. During the treatment of SMAS, if oral ingestion is possible, a small meal is frequently given as the first step of treatment [8]; furthermore, our observations suggest that the transgastric administration of Terumeal uplead® may have a similar effect. This method may be considered useful if gastric fistula is present at the time of treatment.

## Conclusions

In conclusion, our patient with SMAS, which developed after the surgery, was administered with an energy-dense, low-volume nutritional food. This case illustrates that energy-dense, low-volume nutritional feed through a gastrostomy tube can be used to treat the symptoms of post-surgical SMAS. With Terumeal uplead®, using a gastrostomy tube, the symptoms were alleviated without exacerbation. Our observations suggest that the administration of Terumeal uplead® may be effective in the presence of gastrostomy during the conservative treatment phase of SMAS. In addition, if it can be consumed orally by SMAS patients, it may be useful as a supplement in case of insufficient intake.
